# Revealing the clinical significance and prognostic value of small nucleolar RNA SNORD31 in hepatocellular carcinoma

**DOI:** 10.1042/BSR20201479

**Published:** 2020-07-22

**Authors:** Yuan Ding, Zhongquan Sun, Sitong Zhang, Qianhui Xu, Liuzhi Zhou, Dongkai Zhou, Yanjie Li, Xin Han, Hao Xu, Yang Bai, Chang Xu, Hao Ding, Yao Ge, Weilin Wang

**Affiliations:** 1 Department of Hepatobiliary and Pancreatic Surgery, The Second Affiliated Hospital, Zhejiang University School of Medicine, Hangzhou 310009, China; 2 Key Laboratory of Precision Diagnosis and Treatment for Hepatobiliary and Pancreatic Tumor of Zhejiang Province, Hangzhou 310009, China; 3 Research Center of Diagnosis and Treatment Technology for Hepatocellular Carcinoma of Zhejiang Province, Hangzhou 310009, China; 4 Clinical Medicine Innovation Center of Precision Diagnosis and Treatment for Hepatobiliary and Pancreatic Disease of Zhejiang University, Hangzhou 310009, China; 5 Clinical Research Center of Hepatobiliary and Pancreatic Diseases of Zhejiang Province, Hangzhou 310009, China

**Keywords:** cancer biomarker, clinical assessment, hepatocellular carcinoma, non-coding RNA, SNORD31

## Abstract

**Background:** For lack of accurate early diagnosis and prognostic assessment, hepatocellular carcinoma (HCC) becomes severe challenge with the fourth cancer-related mortality. Recently, non-coding RNA (ncRNA) was identified to make functions in progression of various tumors. Among that, a novel ncRNA, small nucleolar RNA C/D box 31 (SNORD31) was suggested in previous study to function as potential tumor suppressing role. In the present study, we aimed to investigate the expression patterns and clinical significance of SNORD31 in HCC.

**Methods:** SNORD31 expression was calculated in HCC cell lines as well as clinical specimens by RT-PCR. HCC patients were subdivided into high and low SNORD31 expression groups and their clinical characteristics were compared. Besides, the association between SNORD31 expression and postoperative prognosis was evaluated using Kaplan–Meier and Cox regression analysis.

**Results:** Compared with corresponding normal reference, expression levels of SNORD31 were significantly down-regulated in both HCC cell lines and clinical specimens (*P*<0.01). Moreover, low SNORD31 expression was remarkably correlated with large tumor diameter, high incidence of vessel carcinoma embolus and capsular invasion, severe tumor differentiation and tumor-node-metastasis (TNM) stage (*P*<0.05). In the following analysis, HCC patients with low SNORD31 expression were independently inclined with poor tumor-free (median time: 9.17 vs 48.8 months, low vs high, *P*<0.001) as well as long-term survival (LTS; median time: 40.26 vs 55.41 months, low vs high, *P*=0.002).

**Conclusions:** The ncRNA SNORD31 was proved to be commonly down-regulated in HCC and was independently associated with multiple malignant characteristics and long-term prognosis of HCC patients, which implied that SNORD31 possessed potential as a novel HCC biomarker.

## Introduction

In the past 20 years, hepatocellular carcinoma (HCC) remains among the top five leading causes of cancer-related deaths all over the world [1]. On one hand, it accounts for the high morbidity of HCC with various predisposing factors, such as hepatitis virus infection, alcohol habituation and metabolic liver diseases [2–4]. On the other hand, for the high rates of recurrence and poor survival of HCC, there is still lack of accurate assessing method and predicting index to be used in individual treatment [5]. Nowadays, although regarded as the most important biomarker for HCC diagnosis, serum α fetal protein (AFP) value are still criticized for its dissatisfactory behavior in the prognostic prediction for HCC patients [6]. Moreover, besides HCC, serum AFP can also be measured to relevantly elevate in other diseases, such as liver fibrosis and reproductive tumors [7]. Thus, there is an urgent need to determine a novel biomarker that is more reliable for the clinical assessment and management of HCC.

Belonging to non-protein-coding RNAs, small nucleolar RNA (snoRNA) plays essential functions in pre-rRNA processing and modification by serving as a guide RNA [8]. According to a previous study, cellular dysregulation of specific snoRNA could result in various physiological and pathological processes [8]. Among that, accumulating evidence has identified that snoRNAs could serve as oncogenes or tumor suppressors in a variety of human tumors [9–11]. Based on these, it is implied that some potential snoRNAs might make a difference in development and progression of HCC.

snoRNA C/D box 31 (SNORD31) is a newly identified snoRNA transcribed from chromosome 11q12.3 genomic region [12]. According to the research of Lucia et al., they determined the abnormal expression patterns of SNORD31 in smoldering myeloma [13]. However, as we know, the underlying roles of SNORD31 in other malignant cancers still remain to be explored yet. Given these above, our present study would investigate the expression features of SNORD31 in HCC. Furthermore, the correlation of SNORD31 expression with clinical significance and the prognostic value in HCC patients was evaluated.

## Materials and methods

### Patient data and tissue specimens

HCC tissues and adjacent liver tissues were acquired from patients who underwent surgical resection between January 2013 and December 2014. Corresponding adjacent tissues were harvested 3 cm from the edges of the tumor lesion. Tissue specimens were immediately put into liquid nitrogen post-operation. The tissues were then stored in a −80°C refrigerator for total RNA extraction. To control the potential confounding factors, all patients were diagnosed with HCC by histopathological examination, while the patients who received chemotherapy or radiotherapy were excluded from the study. All participants signed written informed consent form.

The individual information and clinical characteristics of enrolled patients were obtained from hospital data system, including age, gender, liver function indicators, AFP, tumor lesion characteristics, pathological differentiation, tumor-node-metastasis (TNM) classification and so on. TNM stages were identified according to the American Joint Committee on Cancer TNM classification.

### Follow-up of tumor-free and long-term survival

After hepatectomy, enrolled patients were followed-up by telephone or questionnaire emails for 5 years. Tumor-free survival (TFS) was defined as the time interval elapsed from initial surgery to the recurrence of HCC, HCC distant metastasis or death from any cause without documentation of a cancer-related event. Long-term survival (LTS) was calculated from the date of the initial surgery until death or the last follow-up. Death of patients was ascertained by reporting from the family and verified by review of public hospital records. Clinical staff who collected all the following-up data of HCC patients were blind to participant status, including the individual information, clinical characteristics and SNORD31 expression data.

### Cell culture

QSG-7701 (human hepatic cell line), Hep 3B (human HCC cell line), SK-HEP-1 (human HCC cell line) and Huh-7 (human HCC cell line) were obtained from our laboratory. These cells were maintained in RPMI-1640 medium containing 10% heat-inactivated FBS, 100 U/ml penicillin/streptomycin at 37°C in a humidified atmosphere containing 5% CO_2_.

### Quantitative real-time PCR analysis

Total RNA was isolated from cells, tumors, and adjacent tissues using TRIzol reagent (Invitrogen, Carlsbad, CA, U.S.A.) according to manufacturer’s instructions, and RNA concentration and purity were analyzed in triplicate by Nanodrop 2000 spectrophotometer (Thermo Scientific Inc., Waltham, MA, U.S.A.). After that, the total RNA was reverse transcribed to cDNA using a cDNA Reverse Transcription Kit (Vazyme, Nanjing, China).

To determine the expression level of SNORD31, cDNA sample was tested with quantitative real-time polymerase chain reaction (qRT-PCR) using kits (Roche, Basel, Switzerland). All samples were tested at least three times. The expression of glyceraldehyde-3-phosphate dehydrogenase (GAPDH) was used as an endogenous control, and relative expression of SNORD31 was calculated by comparative *C*_t_ method formula 2^−ΔΔ*C*_t_^. The sequences of all PCR primers used were as follows (5′–3′): SNORD31: CACCAGTGATGAGTTGAATTACCG (forward), ACAGCTCAGAAAATACCTTTCAGTC (reverse); GAPDH: CAGGAGGCATTGCTGATGAT (forward), GAAGGCTGGGGCTCATTT (reverse).

### Statistical analysis

All statistical analyses were performed using the SPSS (Statistical Package for the Social Sciences) 19.0 (SPSS, Chicago, IL). The significance of differences between two cell line or tissue subgroups were estimated by Student’s *t* test. The correlations between SNORD31 expression level and clinical characteristics were analyzed by Chi square test. With use of Kaplan–Meier method, TFS and LTS survival curves were estimated. In the following univariate and multivariate analyses of Cox’s proportional hazard model, independent prognostic indicators were determined. Difference are considered significant for *P*<0.05 and values are presented as standard deviation or number (percentage).

## Results

### The expression patterns of SNORD31 in HCC

**Figure 1:**
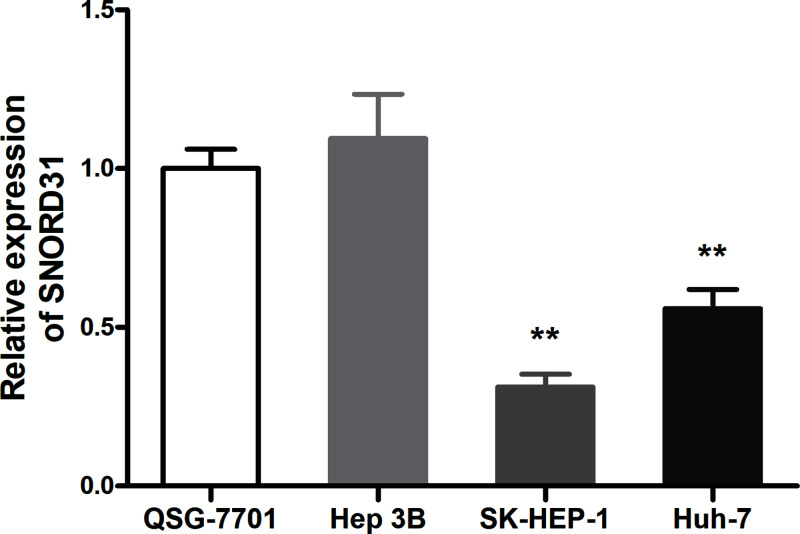
Comparison of SNORD31 expression in cell lines Compared to normal liver cell line QSG-7701, SNORD31 was significantly suppressed in HCC cell lines (SK-HEP-1, *P*=0.001; Huh-7, *P*=0.007). **P<0.01, ***P<0.001.

Compared with QSG-7701 (human hepatic cell line), the SNORD31 expression in SK-HEP-1 as well as Huh-7 (human HCC cell line) were significantly lower (*P*<0.01, Figure 1). This finding indicated that potential dysregulation of SNORD31 might occur in HCC cells.

**Figure 2:**
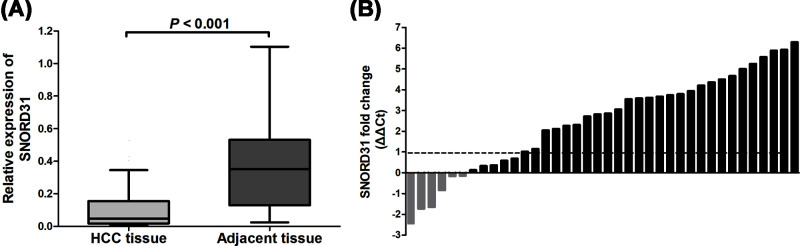
Relative expression of SNORD31 in clinical HCC and adjacent normal tissues (**A**) SNORD31 expression was significantly lower in HCC tissues than that in adjacent normal tissues (*P*<0.001). (**B**) Waterfall plot showed the fold change of SNORD31 expression. ΔΔ*C*_t_ = (*C*_t_ SNORD31 − *C*_t_ GAPDH) of HCC − (*C*_t_ SNORD31 − *C*_t_ GAPDH) of adjacent tissue.

To ascertain this expression patterns of SNORD31, further confirmatory experiment was performed with use of 38 pairs of clinical HCC and adjacent liver tissues. As showed in Figure 2, it showed that SNORD31 was commonly down-regulated in 84.2% HCC specimens compared with corresponding adjacent liver tissues (*P*<0.001). These consistent results above suggested that SNORD31 could play a tumor suppressor role in HCC.

### Association between SNORD31 expression and clinical characteristics of HCC patients

To explore the underlying clinical significance of SNORD31 down-expression of in HCC, all enrolled 137 HCC patients were divided into SNORD31 low expression group (*n*=68) and high expression group (*n*=69) according to individual SNORD31 expression values. Then, various clinical-pathological features were compared between two subgroups under Chi square test. As shown in Table 1, we observed that low SNORD31 expression level significantly correlated with large tumor diameter (*P*=0.041), high incidence of vessel carcinoma embolus (*P*=0.016) and capsular invasion (*P*=0.036), severe tumor differentiation (*P*=0.016) and TNM stage (*P*=0.025). However, SNORD31 expression level was not found to be associated with patients’ age, gender, hepatitis virus B infection, liver function and other indexes (*P*>0.05, Table 1).

**Table 1:** Association of SNORD31 expression and clinical characteristics of HCC patients

Characteristics	Total	SNORD31 expression		*P*-value
		Low (*n*=68)	High (*n*=69)	
Age, years				0.215
<60	88	40 (0.588)	48 (0.696)	
≥60	49	28 (0.412)	21 (0.304)	
Gender				0.075
Female	18	5 (0.074)	13 (0.188)	
Male	119	63 (0.926)	56 (0.812)	
Hepatitis B				0.801
Positive	119	60 (0.882)	59 (0.855)	
Negative	18	8 (0.118)	10 (0.145)	
Cirrhosis				1.000
Present	86	43 (0.632)	43 (0.623)	
Absent	51	25 (0.368)	26 (0.377)	
AFP (ng/l)				0.477
<400	88	46 (0.676)	42 (0.609)	
≥400	49	22 (0.324)	27 (0.391)	
Tumor diameter				0.041*
<5 cm	42	15 (0.221)	27 (0.391)	
≥5 cm	95	53 (0.779)	42 (0.609)	
Multiple lesions				0.649
Present	22	12 (0.176)	10 (0.145)	
Absent	115	56 (0.824)	59 (0.855)	
Vessel carcinoma embolus				0.016*
Present	32	22 (0.324)	10 (0.145)	
Absent	105	46 (0.676)	59 (0.855)	
Microvascular invasion				1.000
Present	9	4 (0.045)	5 (0.072)	
Absent	128	64 (0.941)	64 (0.928)	
Capsular invasion				0.036*
Present	54	33 (0.485)	21 (0.304)	
Absent	83	35 (0.515)	48 (0.696)	
Differentiation				0.016*
Low	74	44 (0.647)	30 (0.435)	
High/Moderate	63	24 (0.353)	39 (0.565)	
TNM stage				0.025*
I–II	97	42 (0.618)	55 (0.797)	
III–IV	40	26 (0.382)	14 (0.203)	

*P<0.05; values are mean ± standard deviation or *n* (%).

### Low SNORD31 expression level indicated poor prognosis in HCC patients

**Figure 3:**
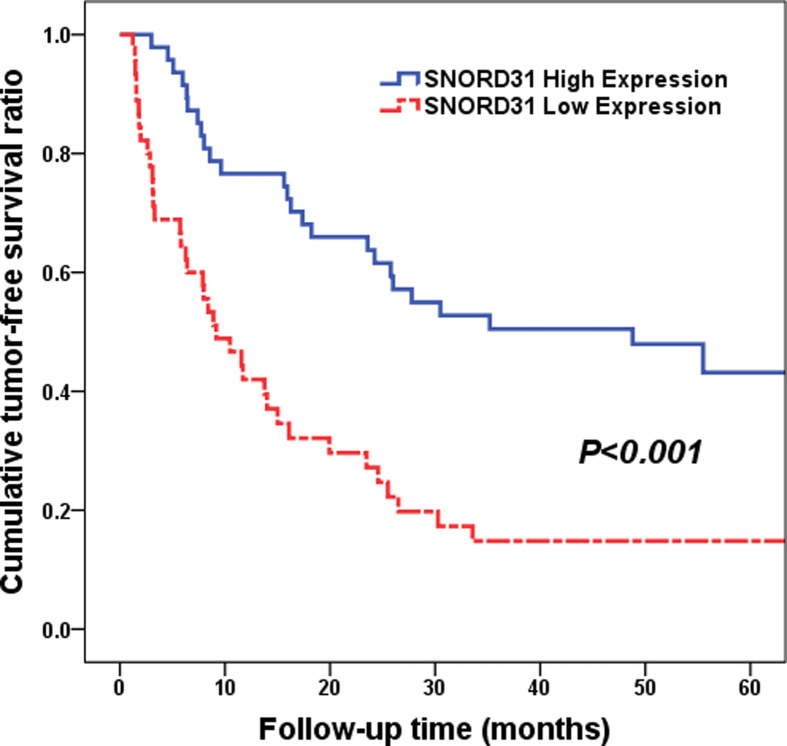
Cumulative TFS ratio between high and low SNORD31 expression subgroups (*P*<0.001) Patients with low SNORD31 expression presented with higher tumor recurrence risk and poorer (median time: 9.17 vs 48.8 months, low vs high, *P*<0.001).

To evaluate the abilities of SNORD31 expression in predicting tumor recurrence and postoperative prognosis for HCC patients, we compared the tumor-free and LTS data between high and low SNORD31 expression subgroups. Under Kaplan–Meier method, patients with low SNORD31 expression presented with higher tumor recurrence risk and poorer (median time: 9.17 vs 48.8 months, low vs high, *P*<0.001, Figure 3). What's more, the following Cox multivariate analysis proved that SNORD31 expression could acted as an independent risk factor for tumor recurrence of HCC patients (hazard ratio (HR) = 0.445, 95% CI: 0.256–0.775, *P*=0.004, shown in Table 2), same as TNM stages (HR = 2.067, 95% CI: 1.218–3.509, *P*=0.007) and vessel carcinoma embolus (HR = 1.872, 95% CI: 1.038–3.375, *P*=0.037).

**Table 2:** Univariate and multivariate analyses of TFS in HCC patients

Clinicopathologic parameters	Univariate analysis		Multivariate analysis	
	HR (95% CI)	*P*	HR (95% CI)	*P*
Age (<60 vs ≥60)	0.708 (0.421–1.192)	0.194		
Gender (Female vs Male)	1.712 (0.737–3.976)	0.211		
Hepatitis B (Negative vs Positive)	1.981 (0.853–4.600)	0.112		
AFP (<400 vs ≥400)	0.848 (0.497–1.446)	0.544		
Cirrhosis (Present vs Absent)	1.351 (0.798–2.286)	0.263		
Microvascular invasion (Present vs Absent)	0.981 (0.356–2.703)	0.970		
Tumor differentiation (Low vs High/Moderate)	1.116 (0.678–1.839)	0.666		
Multiple lesions (Present vs Absent)	1.537 (0.834–2.835)	0.169		
Capsular invasion (Present vs Absent)	1.758 (1.051–2.939)	0.031	1.367 (0.808–2.314)	0.244
Tumor diameter (<5 vs ≥5 cm)	1.655 (0.954–2.870)	0.073	1.386 (0.747–2.572)	0.301
Vessel carcinoma embolus (Present vs Absent)	2.613 (1.507–4.530)	0.001	1.872 (1.038–3.375)	0.037*
TNM stage (I–II vs III–IV)	2.194 (1.304–3.693)	0.003	2.067 (1.218–3.509)	0.007*
SNORD31 expression (Low vs High)	0.360 (0.215–0.602)	<0.001	0.445 (0.256–0.775)	0.004*

*P<0.05 was considered statistically significant.

**Figure 4:**
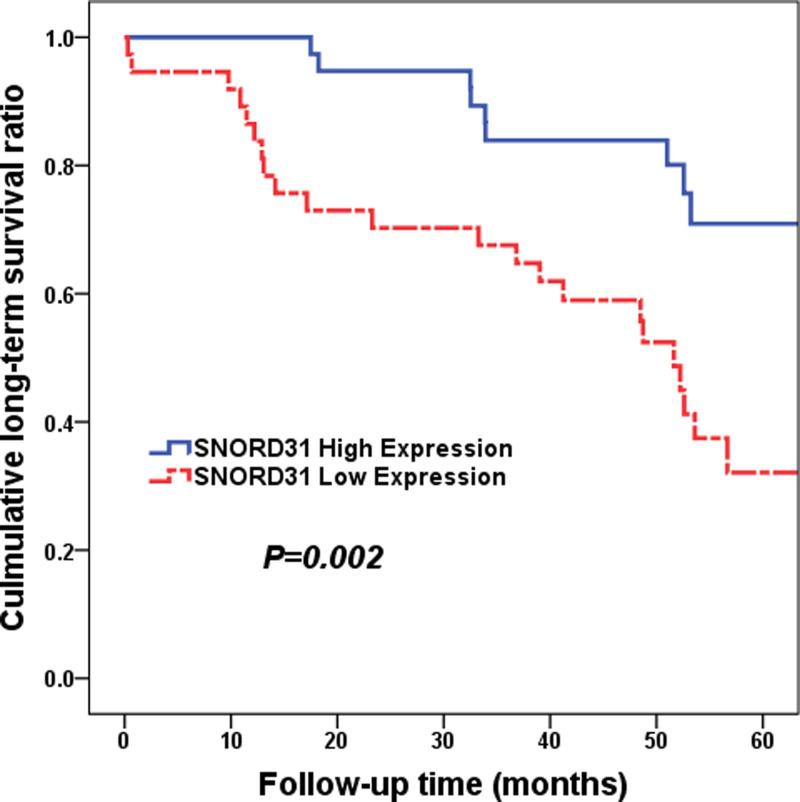
Cumulative LTS ratio between high and low SNORD31 expression subgroups (*P*=0.002) HCC patients with high SNORD31 expression level were commonly inclined to have favorable LTS (median time: 40.26 vs 55.41 months, low vs high, *P*=0.002).

As for the LTS, the same statistics analyzing methods were performed. As shown in Figure 4, HCC patients with high SNORD31 expression level were commonly inclined to have favorable LTS (median time: 40.26 vs 55.41 months, low vs high, *P*=0.002). And then, SNORD31 expression (HR = 0.342, 95% CI: 0.157–0.747, *P*=0.007) as well as TNM stage (HR = 3.905, 95% CI: 1.847–8.258, *P*<0.001) were both shown to be an independent prognostic factor for LTS (Table 3).

**Table 3:** Univariate and multivariate analyses of LTS in HCC patients

Clinicopathologic parameters	Univariate analysis		Multivariate analysis	
	HR (95% CI)	*P*	HR (95% CI)	*P*
Age (<60 vs ≥60)	1.025 (0.502–2.092)	0.946		
Gender (Female vs Male)	0.676 (0.256–1.784)	0.429		
Hepatitis B (Negative vs Positive)	0.902 (0.370–2.202)	0.821		
AFP (<400 vs ≥400)	1.348 (0.664–2.737)	0.408		
Cirrhosis (Present vs Absent)	1.197 (0.573–2.499)	0.632		
Microvascular invasion (Present vs Absent)	1.012 (0.241–4.254)	0.986		
Tumor differentiation (Low vs High/Moderate)	0.860 (0.414–1.787)	0.687		
Capsular invasion (Present vs Absent)	2.187 (1.077–4.441)	0.030	1.700 (0.809–3.572)	0.161
Tumor diameter (<5 vs ≥5 cm)	3.775 (1.318–10.811)	0.013	2.293 (0.741–7.093)	0.150
Multiple lesions (Present vs Absent)	2.017 (0.923–4.408)	0.078	0.806 (0.300–2.161)	0.668
Vessel carcinoma embolus (Present vs Absent)	2.529 (1.188–5.384)	0.016	1.797 (0.820–3.937)	0.143
TNM stage (I–II vs III–IV)	4.191 (2.010–8.739)	<0.001	3.905 (1.847–8.258)	<0.001*
SNORD31 expression (Low vs High)	0.312 (0.144–0.678)	0.003	0.342 (0.157–0.747)	0.007*

*P<0.05 was considered statistically significant.

## Discussion

Due to high rates of metastasis and frequent postoperative recurrence, HCC is recognized as one of the most malignant disease with poor prognosis [14]. HCC in early stage can be effectively treated with curative surgery and liver transplantation, while for advanced cases, the therapeutic strategies are limited [15,16]. Tumor relapse after therapy, as well as drug resistance are the critical issues leading to poor prognosis [16,17]. Despite several clinical variables have been used as the standard for assessing the prognosis of HCC patients, this classification scheme is not precise enough to provide insight into the clinical outcome and therapeutic strategies for individual HCC patient [18,19]. Among that, for example, serum AFP level can be high in some non-cancerous liver disease while sometimes remains at a low level in definite HCC patients [5]. Although liver biopsy can help to reveal tumor biology, it is not used routinely owing to its invasiveness and risk of tumor seeding, especially in early stage patients [19]. Therefore, to explore advanced and promising molecular biomarkers, it is necessary for efficacy of the clinical assessment for HCC patients.

Recent study has indicated snoRNAs play a crucial role in a variety of processes of HCC development, including tumorigenesis, tumor proliferation and metastasis [20,21]. For instance, Han et al.’s study reported that snoRNA SNHGs family, such as SNHG1, SNHG6, SNHG16 and SNHG20, could play varied roles in HCC progression through different regulatory mechanisms by promoting and inhibiting tumorigenesis. Besides, SNORD76 can regulate the development of HCC cell by modulating epithelial-mesenchymal transition (EMT) and Wnt/β-catenin signaling pathway [22]. More and more researches have focused on the function channels of snoRNAs in HCC.

As a newly identified snoRNA, small nucleolar RNA C/D box 31 (SNORD31) is found transcribed from chromosome 11q12.3 genomic region [12]. Indeed, there is rarely studies on the biological or clinical correlation between SNORD31 expression and malignant tumors. Some previous study demonstrated that abnormal expression patterns of SNORD31 was found in smoldering myeloma, while the specific significance of it remained to be explored [13].

In the present study, we for the first time explored the expression features of SNORD31 in HCC and then confirmed the underlying clinical significance. The present study showed that SNORD31 were down-regulated in HCC tissues compared to that in their normal counterparts, and SNORD31 expression level was remarkably associated with tumor diameter, vessel carcinoma embolus, capsular invasion, tumor differentiation and TNM stage. These results above suggested that SNORD31 expression level was correlated with malignant behaviors of HCC, including tumorigenesis, invasion and metastasis. It was suggested that SNORD31 might play crucial roles in the development and progression of HCC. Our finding indicated that patients with higher expression level of SNORD31 in HCC specimens had a better TFS and LTS than patients with lower expression, suggesting that low expression of SNORD31 in HCC probably facilitate an increased malignant and worse prognostic phenotype. In addition, Cox multivariate analysis showed that low expression level of SNORD31 served as an independent risk factor in unfavorable survival and worse prognosis in HCC patients. As far as we know, this was the first study to propose the clinical significance of SNORD31 expression in predicting the TRS and LTS of patients with HCC. However, further investigations are needed to illuminate the detailed molecular mechanisms by which SNORD31 plays regulatory roles in HCC.

Taken together, our study revealed that SNORD31 expression was down-regulated in HCC and was closely associated with advanced HCC progression. Furthermore, SNORD31 expression was demonstrated for the first time to be a potential independent biomarker to predict the prognosis of HCC patients. Thus, the present study helped in the discovery of novel biomarker of HCC, and even would lead to the new targets for effective therapy of human HCC in the future.

## Data Availability

The datasets used and/or analyzed during the current study are available from the corresponding author on reasonable request.

## Abbreviations

AFP: α fetal protein
HCC: hepatocellular carcinoma
HR: hazard ratio
LTS: long-term survival
SNORD31: small nucleolar RNA C/D box 31
snoRNA: small nucleolar RNA
SPSS: Statistical Package for the Social Sciences
TFS: tumor-free survival
TNM: tumor-node-metastasis
